# Use of a Serious Game to Teach Infectious Disease Management in Medical School: Effectiveness and Transfer to a Clinical Examination

**DOI:** 10.3389/fmed.2022.863764

**Published:** 2022-04-25

**Authors:** Alexandra Aster, Simone Scheithauer, Angélina Charline Middeke, Simon Zegota, Sigrid Clauberg, Tanja Artelt, Nikolai Schuelper, Tobias Raupach

**Affiliations:** ^1^Institute of Medical Education, University Hospital Bonn, Bonn, Germany; ^2^Institute for Infection Control and Infectious Diseases, University Medical Center Göttingen, Göttingen, Germany; ^3^Department of Cardiology and Pneumology, University Medical Center Göttingen, Göttingen, Germany; ^4^Medius KLINIK Ostfildern-Ruit, Ostfildern-Ruit, Germany

**Keywords:** medical education, teaching, serious game, objective structured clinical examination, Item Response Theory

## Abstract

**Purpose:**

Physicians of all specialties must be familiar with important principles of infectious diseases, but curricular time for this content is limited and clinical teaching requires considerable resources in terms of available patients and teachers. Serious games are scalable interventions that can help standardize teaching. This study assessed whether knowledge and skills acquired in a serious game translate to better performance in a clinical examination.

**Methods:**

Fifth-year undergraduate medical students (*n* = 100) at Goettingen Medical School were randomized to three groups receiving different levels of exposure to virtual patients presenting with signs and symptoms of either infective endocarditis or community-acquired pneumonia in a serious game simulating an accident and emergency department. Student performance was assessed based on game logfiles and an objective standardized clinical examination (OSCE).

**Results:**

Higher exposure to virtual patients in the serious game did not result in superior OSCE scores. However, there was good agreement between student performance in the OSCE and in game logfiles (*r* = 0.477, *p* = 0.005). An Item Response Theory analysis suggested that items from the serious game covered a wider range of ability, thus better differentiating between students within a given cohort.

**Conclusion:**

Repeated exposure to virtual patients with infectious diseases in a serious game did not directly impact on exam performance but game logfiles might be good and resource-sparing indicators of student ability. One advantage of using serious games in medical education is standardized content, a lower inhibition threshold to learn, and a need of less staff time compared to small-group clinical teaching.

## Introduction

Learning how to manage infectious diseases is an important goal of undergraduate medical education. Patient management encompasses the application of relevant knowledge to a particular situation. The process of organizing the available information, formulating a differential diagnosis, ordering appropriate tests and drawing adequate therapeutic consequences from their results is called clinical reasoning (1). Case-based learning in small groups is one approach to enhancing clinical reasoning skills in medical students, but it requires considerable resources in terms of time and staff. Likewise, procedural knowledge can hardly be tested in multiple choice questions. Thus, comprehensive assessment formats such as objective standardized clinical examinations (OSCE) have been introduced, but these are time-consuming as well. Additionally, not all departments of infection control and/or infectious diseases are adequately staffed to offer interactive teaching in small groups and run OSCE stations related to their field of expertise. Thus, innovative teaching and assessment formats are needed.

Serious gaming as an operationalisation of game-based learning is an innovative approach that can be rooted in part in self-determination theory. A number of studies suggest that undergraduate medical students may benefit from using serious games in terms of superior learning outcome. For example, a virtual accident and emergency (A&E) department has been shown to be non-inferior to traditional problem-based learning (2). One prospective trial with a non-exposed control group reported a sustained and superior learning outcome with regard to clinical reasoning even after 1.5 years of follow-up (3). Moreover, learning outcome with this instructional format may not be entirely case-specific, i.e., the procedural knowlegde acquired through playing the game can be transferred to new cases (4).

In particular, since infectious diseases and infection prevention are cross-sectional areas, which in some cases are addressed only incidentally in practical training and not as a core topic of the respective subject, this innovative and a priori efficient and scalable approach could represent a solution for the challenges in teaching outlined above–provided it is effective.

One important limitation of studies assessing the effectiveness of serious games in medical education is that most of them lack objective outcome data and merely focus on descriptions of the resources used (5). While key feature questions lend themselves to the assessment of clinical reasoning skills (6), transfer of skills demonstrated in these written examinations into the real world of patient care is at least questionable. In order to know whether the use of serious games in undergraduate medical education impacts the quality of care, demonstration of adequate skills in an OSCE would be a useful first step. Given the effort associated with running an OSCE, it would be interesting if gaming scores can be used as a surrogate marker of student learning outcome so that parts of the examination could be replaced by a summative gaming session.

To date, very few studies have assessed the impact of learning with serious games on performance in clinical practice (7).

The first aim of this study was to investigate the association between clinical reasoning training through exposure to virtual infectious disease (ID) patient cases in a serious game simulating a digital A&E department and student performance in an OSCE designed to assess ID patient management skills. We hypothesized that increased exposure to similar virtual ID patients in the serious game would produce higher performance levels in the ID-related OSCE.

The second aim of the study was to compare item characteristics between game logfiles and OSCE checklists in order to determine whether the game itself can be used as an assessment tool instead of or in addition to an OSCE. Thus, it would add an effective and efficient tool for assessing competence improvement also suitable for situations in which personal contacts are restricted.

## Methods

### Study Design

In winter term 2019/2020, fifth-year undergraduate medical students who were enrolled in a 6-week repetition module at Goettingen Medical School were invited to participate in this monocentric, prospective, randomized trial. Students were required to take 6 weekly 90-min teaching sessions with the virtual A&E department. During all sessions, students worked on virtual patient cases in a self-directed manner and at their own pace, without any formal teaching. In total, all students were exposed to up to 45 different cases presenting with various symptoms and relating either to internal medicine or to neurology:

Session 1: Hyponatriemia, non-cardiac chest pain, silent myocardial infarction, chronic obstructive pulmonary disease, acute renal failure, anaphylaxis.
Session 2: stable pulmonary embolism, hypoglycaemia, epileptic seizure, gastroenteritis, pleural effusion, drug intoxication, first presentation of chronic lymphatic leukemia.
Session 3: Hodgkin's Disease, congestive heart failure, appendicitis, compartment syndrome, diabetic ketoacidosis, diverticulitis, hypertensive crisis.
Session 4: upper gastrointestinal bleed, acute urinary retention, rhabdomyolysis, pernicious anemia, febrile spasm, acute back pain, urogenital tuberculosis.
Session 5: pseudocroup, hypokalaemia, alcohol intoxication, erysipelas, hyponatraemia, myocardial infarction, chronic obstructive pulmonary disease.
Session 6: subarachnoidal bleed, erysipelas, multiple sclerosis, shoulder dislocation, sarcoidosis, abscess.

At the beginning of term, students were stratified by sex and previous performance levels and subsequently randomized to 1 of 3 groups (A, B, AB). During the 6-week module, groups A and B were presented with one additional specific intervention case each. Thus, group A was exposed to the specific intervention case ‘community-acquired pneumonia' (CAP) twice during the intervention phase (at session 3 and session 5), while group B was exposed to the specific intervention case “infectious endocarditis” (EC) (at session 3 and session 5). Group AB was presented with both specific intervention cases, but only once (at session 4). Thus, all groups used the serious game but exposure to specific virtual patient cases differed between groups.

The CAP case was a virtual patient of either gender aged between 65 and 85 years who presented with a cough and phlegm. Their respiratory rate was set to 35/min, their body temperature to between 38.0 and 39.7°C, their heart rate to between 80 and 110/min, and their oxygen saturation to 85–90%. The history as well as findings on physical examination and a chest X-ray were in line with the diagnosis of community-acquired pneumonia.

The EC case was a female virtual patient aged between 60 and 80 years presenting with a fever of 39.5°C that had occurred repeatedly over the course of the past 2 weeks. The physical examination revealed splinter hemorrhages as well as a systolic murmur in the mitral region. Cardiac ultrasound was suggestive of infective endocarditis.

Irrespective of study participation, all students took a summative OSCE at the end of the six-week repetition module. Passing the OSCE was a prerequisite for obtaining a final-year placement at Goettingen Medical School. The OSCE was composed of eight stations each covering complex clinical-practical skills, such as taking a history, carrying out a physical examination, and interpreting examination and test findings. Two of these stations were aligned to the intervention cases shown in the serious game. Performance raters were two senior physicians who had been specifically trained to judge student performance on checklists containing 15 (EC) or 16 (CAP) items, respectively (see Supplementary Table 1 for checklists). Student performance on the same items during gaming sessions were logged and used for a comparatory analysis.

### Intervention

In this study, we used serious game simulating an A&E department. In the game, each student directed a physician avatar through a 3D simulation of an A&E ward, triaging and treating up to ten patients with different diseases simultaneously. Tasks included taking a history, choosing appropriate diagnostic tests, identifying the most likely diagnoses and taking adequate therapeutic measures with many of such activities having an immediate effect on the patient's vital signs. Upon completion of each case, students received a digital feedback on the patient's treatment as well as a (virtual) senior physician's recommendation on how to successfully solve the case. Screenshots of the game have been published elsewhere (2). The gamification elements used in the serious game included the avatar representing the student player, the plot prompting students to look after their patients, the time pressure created by the arrival of new patients and the threat of deterioration if important measures were delayed; and finally a performance graph outlining each student's actions on a timeline and a table comparing their activities to an ideal management plan for a specific patient. While using the computer-based A&E department, all gaming activities were automatically saved in logfiles.

### Student Enrolment, Data Collection and Statistical Analysis

Before the start of the module, students were informed about the study by e-mail. On the first day of the 6-week repetition module, the study rationale was explained in detail and students were invited to provide written consent to participate in the study and to have their data analyzed. The local Institutional Review Board (application number 11/8/19) waived ethics approval as the study protocol was not deemed to represent biomedical or epidemiological research. We made every effort to comply with data protection rules and all data were anonymized prior to analysis. Study participation was voluntary.

For the analysis of student performance while playing the serious game, logfiles that were generated during sessions were examined. For each of the two intervention cases, appropriate actions were defined according to current guidelines and their locally adopted recommendations (8–11). In the EC case, five points were awarded for relevant aspects of patient history, two points for examinations and tests, three each for appropriate laboratory and microbiological tests and appropriate antibiotic treatments, and one each for the correct diagnosis and transfer to the appropriate ward. In the CAP case, there were four points for aspects of patient history, three for patient examination and tests, six for laboratory and microbiological tests and one each for the correct diagnosis, correct treatment and appropriate patient allocation.

OSCE checklists contained identical items, and scores were computed according to the description above. Aggregate scores across all items were computed, and most of the analyses were conducted using these scores. In addition, the normal distribution of the underlying category scores was assessed using the Shapiro-Wilk test. This revealed that only the total sum scores (and not the category subscores) were normally distributed, thus non-parametric methods were used to further analyse the data.

Logfile and OSCE data were added to a database also containing information on student sex and age as well as prior performance levels in summative examinations. Internal consistency of in-game activity (as assessed by logfiles) and OSCE checklists was examined by means of Cronbach's alpha.

#### Analysis for Study Aim 1

The association between exposure intensity in the virtual A&E department (once or twice per case) and learning outcome in the same cases was assessed by comparing OSCE scores by case across the three groups using a Wilcoxon Test comparing central tendencies. Performance increases between the first and second exposure to the CAP case (group A) and the EC case (group B) were assessed by means of an ANOVA. Even in situations where data are not normally distributed, the ANOVA is robust against violations of the normal distribution assumption provided that the groups are about the same size (12). Given that this was the case in this study, an ANOVA with repeated measurements with *post-hoc* paired *T* tests (if indicated) was conducted.

#### Analysis for Study Aim 2

Agreement between logfile scores in the last occurrence of a specific virtual patient case and OSCE scores for the same disease was assessed by means of a Pearson correlation and a Bland Altman Plot. Finally, both logfile and OSCE checklist items were characterized according to Item-Response Theory in order to assess which of the two was more suitable to differentiate between students at various competency levels.

Statistical analyses were performed using IBM SPSS Statistics 25.0 (IBM Corp, Armonk, NY). Significance levels were set to 5%.

## Results

### Participant Characteristics and Assessment Quality

**Figure 1:**
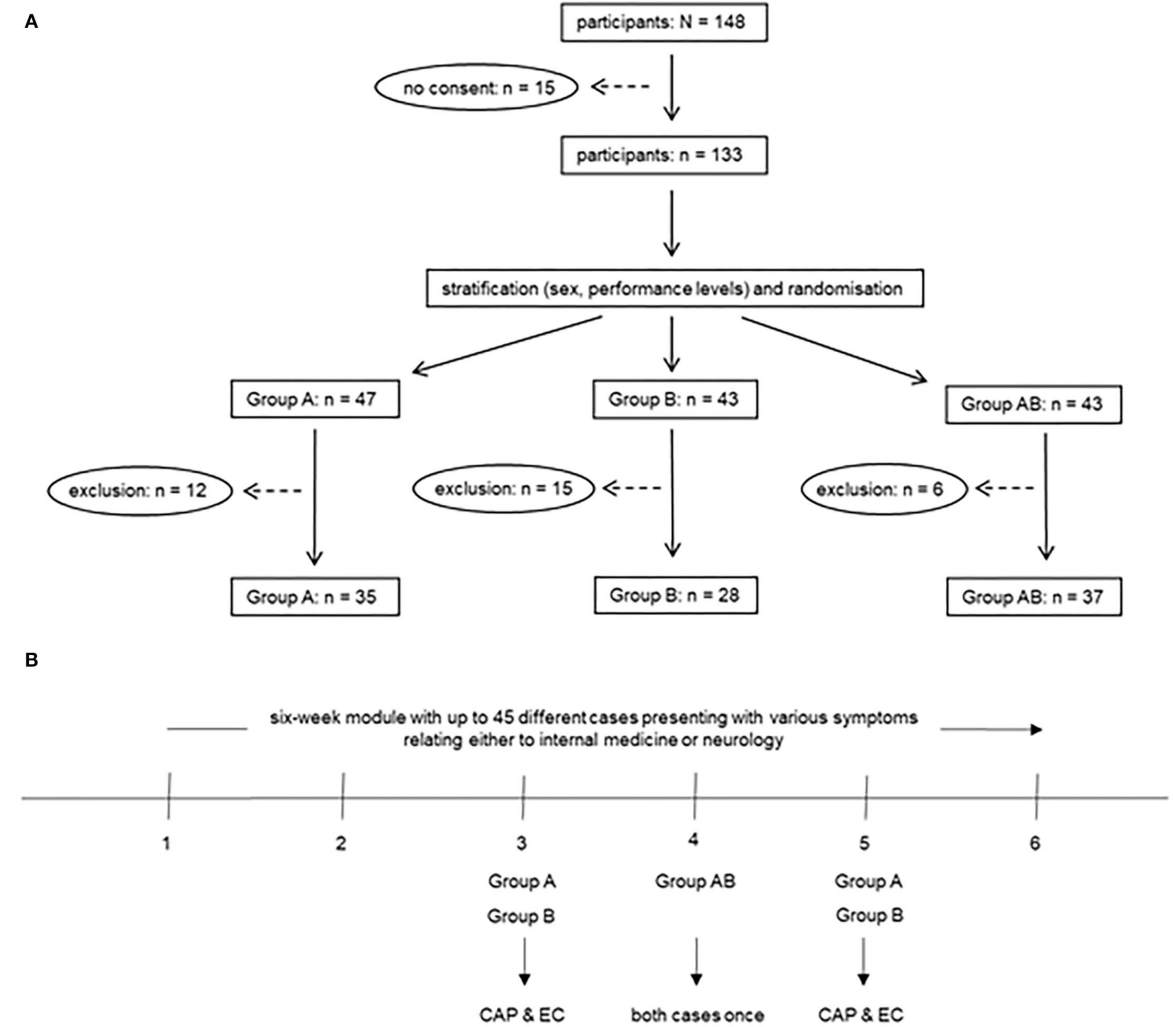
**(A)** Flow of participants through the study. Exclusion was due to non-compliance with the study protocol (e.g., when students were erroneously exposed to the wrong cases at least once because they did not attend the session they had been assigned to but participated in a different session). **(B)** Time course of the study.

A total of 148 students were eligible for study participation, 15 of whom did not provide written consent. After exclusion of another 33 students due to non-compliance with the study protocol (e.g., attendance at a session they had not been assigned to and therefore at least one exposure to the wrong cases), complete data were available for 35, 28 and 37 students in groups A, B and AB (Figure 1). The effective response rate was 67.6% (100 out of 148 eligible students). A total of 77.0% (*n* = 77 out of a population of *N* = 100) of the participants were female. There were no significant between-group differences regarding age, sex distribution and examination scores during the previous semester (Table 1). Cronbach's α values for game logfiles and OSCE checklists were 0.725 and 0.352 for the EC case and 0.641 and 0.200 for the CAP case, respectively. In terms of OSCE performance, the mean total percent score for endocarditis was highest for Group A (79.7%), followed by Group B (78.0%) and Group AB (74.3%). In contrast, Group B (68.0%) had the highest mean total OSCE score for CAP, followed by Group A (62.7%) and Group AB (60.8%).

**Table 1:** Participant characteristics.

	**A (*n* = 35)**	**B (*n* = 28)**	**AB (*n* = 37)**	***p*-value**
Age [years]	25.7 ± 2,3	26.0 ± 3.5	25.5 ± 2.9	0.536
Female [%]	65.7	67.9	67.6	0.918
Examination scores during the previous semester [%]	87.5 ± 4.7	88.4 ± 4.9	86.1 ± 5.2	0.174

*P-values were derived from a Kruskal-Wallis-Test or a χ^2^ test, as appropriate*.

### Study Aim 1: Association Between Exposure and Learning Outcome

Regarding the CAP case, running an ANOVA was possible despite the data not being normally distributed. There was a significant between-group difference (*F*_(2, 99)_ = 3.922, *p* = 0.023, $\eta_{\text{p}}^{2}$ = 0.073). About 5% of the variance of OSCE scores could be explained by the attendance at repeated gaming sessions (effect size f = 0.29; equivalent to a medium effect). Bonferroni corrected multiple comparisons showed a significant difference (*p* = 0.018) between group B (10.8 ± 1.7 points) and group AB (9.7 ± 1.7 points). There were no significant differences between group A (10.2 ± 1.5 points) and group B (*p* = 0.355) or between group A and group AB (*p* = 0.628).

Regarding the EC case, the ANOVA pre-requisite of variance homogenity was not met. Therefore, a Welch-ANOVA was conducted, and this revealed no significant group differences (*F*_(2, 65.166)_ = 0.382, *p* = 0.684).

### Performance Increase Between Two Gaming Sessions

**Figure 2:**
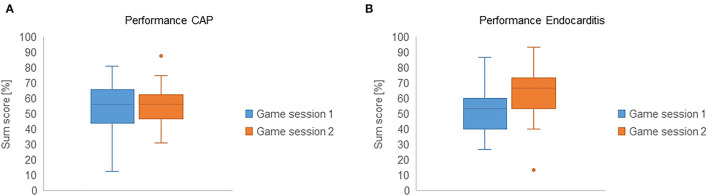
Boxplots of logfile sum scores (%) in students who completed the same case twice. **(A)** CAP (group A); **(B)** EC (group B).

Concerning the CAP case, there was no significant increase between the two gaming sessions (median = 9 & 9, Wilcoxon signed-rank test: z = −0.486, *p* = 0.627, *n* = 35, effect size *r* = 0.08, *R*^2^ = 0.007). In contrast, the increase between the two sessions was significant in the EC case (median = 7 & 10, Wilcoxon signed-rank test: z = −3.008, *p* = 0.003, *n* = 28, effect size r = 0.57, *R*^2^ = 0.323; see Figure 2). Both the effect size and the coefficient of determination corresponded to a large effect.

### Study Aim 2: Agreement Between Game Logfiles and OSCE Checklists

The correlations between the last respective gaming session and the OSCE rating for a specific case revealed a significant positive effect of medium strength for EC (*r* = 0.477, *p* = 0.005), whereas there was no significant correlation for CAP (*r* = 0.121, *p* = 0.244). In general, OSCE performance was higher than in-game performance for both cases.

**Figure 3:**
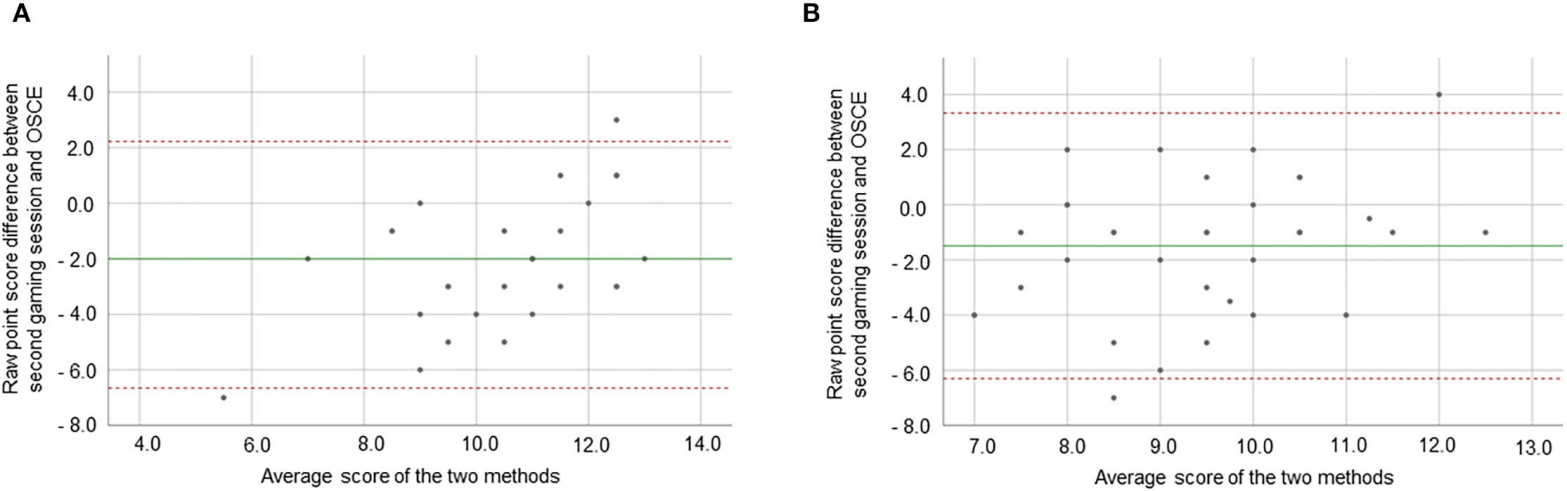
Agreement between total scores in game logfiles and OSCE checklists for the two cases. **(A,B)** Bland Altman Plots for the EC and CAP case, respectively.

The Bland-Altman Plots did not indicate systematic biases. Figure 3 illustrates that the size of differences between the two measurement types (logfiles / OSCE checklists) was acceptable.

### Exploratory Analysis According to Item Response Theory

**Figure 4:**
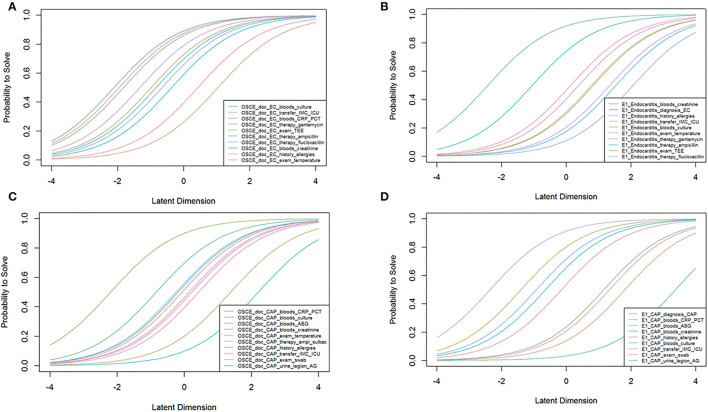
Analyses according to Item Response Theory. Graphs indicate that the serious game is more suitable to differentiate between students within a given cohort, as those items cover a wider range of the underlying student ability. The further to the right on the X-axis the inflection point of the curve is located, the higher the person's ability level must be in order to be able to solve the item with a 50 percent probability. **(A)** EC case (OSCE); **(B)** EC case (game); **(C)** CAP case (OSCE); **(D)** CAP case (game).

In order to assess which of the two assessments methods is better suited to differentiate between students with more or less favorable clinical reasoning skills, analyses according to Item Response Theory was conducted. In order to assure good model fit, some items had to be excluded from IRT analyses; thus, the Item Characteristic Curves displayed in Figure 4 were based on slightly different item subsets for game logfiles and OSCE checklists, respectively. More precisely, items were excluded due to their lacking capability to measure the underlying abilites of the students, as the items were classified as being too easy or too difficult to solve prior to the analysis. The graphs indicate that OSCE checklists tend to cover lower ability ranges than game logfiles, especially in the case of CAP. In addition, game logfile items cover a broader spectrum while many OSCE checklist items appear to predominantly cover lower or medium ability levels (around−1 Logit in EC and 0 in CAP). A detailed analysis of item difficulty and discriminatory power according to classical test theory as well as item difficulty and item correlation according to IRT is provided in Supplementary Table 1 the Online Supplement.

## Discussion

This monocentric, prospective study investigated the impact of using a serious game on performance in a clinical-practical examination in the context of infectious disease teaching. The first aim of this study was to assess the association between exposure to the virtual A&E department and student performance in an OSCE designed to assess infectious disease patient management skills. We hypothesized that more intensive exposure to similar virtual ID patients in the serious game would result in higher performance levels in the case-related OSCE station. While there were no significant differences in OSCE scores between cases with higher or lower previous exposure, the results presented in Figure 2 suggest that repeated exposure to the infective endocarditis case increased student performance in that particular case.

The second aim of the study was to compare item characteristics between game logfiles and OSCE checklists in order to determine whether the game itself can be used as an assessment tool instead of or in addition to an OSCE. According to the Bland Altman Plots in Figure 3, agreement between total scores derived from game logfiles and OSCE checklists was acceptable. We also chose an Item Response Theory approach to address this question as these analyses provide information over and above what can be inferred from classical test theory. The data indicated that many of the OSCE checklist items covered the same student ability level (i.e., around the median of the entire cohort in the infective EC case and around−1 Logit in the CAP case). This means that the OSCE is less suitable to differentiate between students at the upper ends of the ability spectrum. In contrast, item characteristic curves for game logfile items yield a wider distribution. In general, this indicates that a test is particularly useful for a student sample with diverse ability levels. Such a sample can be assumed when a newly learned skill is tested and both low and high ability level students should be able to solve some of the tasks–as was the case in this study. Based on our findings, the serious game appears to be better suited than the OSCE to differentiate between students with a higher underlying ability. It can be assumed that this ability could be clinical reasoning.

Apart from these theoretical considerations, it is interesting to see that both in the OSCE and the game, the item referring to the Legionella urinary antigen test was useful in differentiating between students even at the upper end of the performance spectrum. In contrast, the item referring to flucloxacillin as part of the regimen for empirical treatment of endocarditis appeared to be more useful in a gaming context to differentiate between very good and excellent students.

### Methodological Issues

OSCE checklists were closely aligned to game logfile items in order to compare the two modalities. Across all three student groups, there was a striking difference between OSCE and logfile scores in that for both diseases, OSCE scores were about 10% higher than scores achieved in the final gaming session before the OSCE. One potential explanation for this finding is that–according to the retrieval hypothesis (13)–playing the game could have enhanced retention, thus leading to more favorable OSCE scores. If this were the case, a similar increase would be expected between the first and the second gaming session in the subsample of students who were exposed to the same disease twice. However, this effect was only observed for the infective endocarditis and not for the CAP case. An explanation for this finding might be the CAP case was less difficult, already internalized or more intuitive for the students. Given that a number of previous studies on repeated testing have used more than two exposures to the same material (14, 15), additional gaming sessions might have been required to detect the anticipated effect.

Despite the similarities between logfile and OSCE checklists, the two formats differed in one important regard. While students play the game individually and without interacting with other people, the very idea of an OSCE is to reproduce the clinical setting in order to facilitate immersion in the future workplace. Students might find it easier to accommodate to an OSCE situation, and retrieval of relevant information might be facilitated in circumstances more resembling the clinical environment because this is where a lot of teaching takes place as well (16, 17). In addition, the behavior of standardized patients in the OSCE might have prompted actions that were not triggered in the gaming context.

A third explanation for the marked difference between logfile and OSCE scores is that the OSCE was a summative examination, i.e., it was marked and students had to pass it in order to get a clinical placement for their subsequent year in medical school. Summative exams are known to have a much stronger impact on student behavior than formative (i.e., non-graded) exams (18). Thus, compared to gaming sessions that were labeled as learning activities, students might have been more motivated to achieve favorable scores in the clincal-practical exam.

### Implications for Teaching and Testing on Infectious Diseases

This was the first study to ever report an IRT analysis of gaming logfiles in comparison with OSCE checklist items. One interesting finding is that logfile items appeared to better differentiate between students of different capability across a wider spectrum than the OSCE. In view of the aspects discussed above, student performance in the game might actually be a more valid surrogate parameter of actual student performance levels [as summative exams cause students to learn to the test, and retention of skills demonstrated in a summative test is usually short-lived (19)]. According to the IRT data, game logfiles facilitated differentiation of students across a wider spectrum of ability levels which might render serious gaming a potential alternative and/or addition to traditional, resource-intensive assessment formats. *One* possible explanation for this might be that serious games offer more flexibility in what can be done. This, however, allows for more errors, which in turn makes it harder to react correctly and lowers the probability of acting according to guideline recommendations. This might actually be positive, as real-life situations are also flexible and not as limited as OSCEs.

With regard to teaching itself, serious games might be advantageous because they enable training and testing independent of time and place, which is not only helpful at times and under the conditions of a pandemic. Furthermore, if curricular integration is ensured, this tool can be scaled up and, in contrast to standard methods, can be a profound, effective alternative or supplement that spares teacher resources. The focus should not only be on students: Serious games can also be used in further medical training (e.g., continuing medical education), and in the context of Antibiotic Stewardship trainings or for physicians in training for infection control link physicians in various departments. Particularly, in light of the COVID-19 pandemic, serious games offer an innovative approach of teaching hospital employees about infection prevention and control (20).

### Strengths and Limitations

This randomized and prospective trial enrolling a fairly large sample of medical students used a complex serious game to teach content that is hard to standardize in clinical teaching. In fact, we did not compare learning in a serious game to learning in the real clinical context because patient encounters in a real accident and emergency ward cannot be standardized. The main outcome was student performance, outcome measures were aligned to teaching objectives, and logfile and OSCE checklist items were closely aligned as well.

The high drop-out rate due to protocol violations and the resulting unequally sized subsamples is the most important limitation of the study, and therefore the interpretation of our results is limited. This has not only led to a reduction of power to detect significant effects, but also to a decreased reliablity which renders generealisation of our findings to a broader sample of students difficult. Nevertheless, this study can be seen as an encouraging starting point for future research. The lack of a significant correlation between logfile and OSCE scores related to the CAP case indicates that the rubrics might need to be further improved. Moreover, given the case-specificity of OSCE stations, a greater number of diseases should be included in order to draw generalisable conclusions on the effectiveness of serious games in infectious diseases teaching. Finally, none of the outcome measures was derived from actual clinical practice. Workplace-based assessments would be needed to generate such data, but the downside of that approach is that exposure to clinical cases in the work environment cannot be standardized to the same extent as in an OSCE.

To our knowledge, IRT has not been used to assess the quality of logfile items before. The approach appears to be promising, but more studies are needed to confirm that game logfiles can be used as a surrogate or an addition to more traditional measures of student performance. For example, it is unclear whether the same trait affects the ability to answer questions in an OSCE setting and in a serious game setting, more precisely, it is unclear whether the OSCE measures only one ability as is necessary for IRT analyses to be valid.

### Conclusions

Greater exposure to virtual patients presenting with signs and symptoms of infectious diseases in a serious game simulating a virtual A&E department did not lead to enhanced performance in an OSCE covering these diseases. The agreement between game logfile and OSCE scores and the results of the IRT analyses suggest that a serious game can generate useful information on student ability and might thus complement other measures of student performance.

## Data Availability Statement

Original data and materials will be shared upon reasonable request.

## Ethics Statement

The studies involving human participants were reviewed and approved by Institutional Review Board at Goettingen Medical School. The patients/participants provided their written informed consent to participate in this study.

## Author Contributions

AA analyzed the data, created figures and tables, and wrote the manuscript. SS wrote parts of the manuscript and designed the infectious disease parts. AM designed logfile checklists as well as OSCE stations, helped to analyse the data, and wrote parts of the first draft of the manuscript. SZ conducted the IRT analysis. SC and TA assisted in terms of infection prevention combined with expertise in student teaching. NS created virtual patient cases for the serious game. TR conceived of the study, developed its design, was involved in data analysis, assisted in the design of OSCE stations, wrote parts of the first manuscript draft, and commented on all subsequent versions. All authors contributed to the article and approved the submitted version.

## Conflict of Interest

The authors declare that the research was conducted in the absence of any commercial or financial relationships that could be construed as a potential conflict of interest.

## Publisher's Note

All claims expressed in this article are solely those of the authors and do not necessarily represent those of their affiliated organizations, or those of the publisher, the editors and the reviewers. Any product that may be evaluated in this article, or claim that may be made by its manufacturer, is not guaranteed or endorsed by the publisher.
