# Supplementary material for: Use of a Serious Game to Teach Infectious Disease Management in Medical School: Effectiveness and Transfer to a Clinical Examination
Source: Front Med (Lausanne). 2022 Apr 25;9:863764. doi: 10.3389/fmed.2022.863764 (PMC9082676; doi:10.3389/fmed.2022.863764)
Supplement: Supplementary file 1 [file Table_1.pdf]

Table S1: Item difficulty and selectivity for parameters of Classical Test Theory (CTT) and Item Response Theory (IRT) regarding all variables for EC as well as for CAP in both the game and the OSCE. CRP, C-reactive protein; PCT, procalcitonin; TOE, transoesophageal echocardiography; ABG, arterial blood gases

	Serious game			OSCE		
	**CTT- item difficulty**	**CTT – selec-tivity**	**IRT-para-meter (SE)**	**CTT-item difficulty**	**CTT- selec-tivity**	**IRT-para-meter (SE)**
**Variables Endocarditis**						
History: Allergies	0.57	-0.029	0.13 (0.22)	0.47	0.019	0.42 (0.17)
Examination: Temperature	0.42	0.172	0.84 (0.23)	0.33	-0.029	1.03 (0.18)
Bloods: CRP or PCT	/	/	/	0.89	0.050	-1.83 (0.25)
Bloods: Creatinin	0.94	0.031	-2.43 (0.42)	0.65	-0.114	-0.37 (0.18)
Investigation: TOE	0.69	0.205	2.06 (0.29)	0.72	0.050	-0.99 (0.20)
Investigation: Blood culture	0.43	0.357	0.78 (0.23)	0.92	0.023	-2.12 (0.28)
Correct diagnosis	0.79	0.341	-1.06 (0.26)	/	/	/
Treatment: Gentamycin	0.25	0.578	1.34 (0.25)	0.83	0.403	-1.33 (0.22)
Treatment: Ampicillin	0.22	0.529	1.53 (0.26)	0.74	0.352	-0.84 (0.19)
Treatment: Flucloxacillin	0.15	0.570	2.06 (0.29)	0.73	0.410	-0.66 (0.18)
Patient transfer	0.48	0.135	0.38 (0.22)	0.91	0.278	-1.96 (0.27)
**Variables Community-acquired pneumonia**						
History: Allergies	0.59	0.038	-0.15 (0.22)	0.57	0.051	0.22 (0.17)
Examination: Temperature	/	/	/	0.56	-0.123	-0.07 (0.17)
Bloods: CRP or PCT	0.89	0.290	-1.40 (0.28)	0.91	0.217	-2.20 (0.29)
Bloods: Creatinin	0.78	0.353	-0.65 (0.23)	0.61	-0.052	-0.19 (0.17)
Bloods: ABG	0.77	0.189	-0.93 (0.24)	0.67	0.199	-0.25 (0.17)
Urine: Legionella antigen	0.04	-0.007	3.37 (0.5)	0.16	0.213	2.20 (0.24)
Investigation: Blood culture	0.30	0.278	1.16 (0.24)	0.68	0.203	-0.84 (0.19)
Investigation: Sputum smear	0.19	0.237	1.81 (0.28)	0.23	-0.043	1.38 (0.19)
Correct diagnosis	0.93	-0.033	-2.36 (0.39)	/	/	/
Treatment: Ampicillin + Sulbactam	/	/	/	0.50	0.040	0.14 (0.17)
Patient transfer	0.26	0.151	1.33 (0.24)	0.45	0.032	0.42 (0.17)

Notes. EC: Cronbach's Alpha for EMERGE: 0.600; Cronbach's Alpha for OSCE: 0.358. CAP: Cronbach's Alpha for EMERGE: 0.418; Cronbach's Alpha for OSCE: 0.335.
